# Antibody response in vaccinated pregnant mares to recent G3BP[12] and G14P[12] equine rotaviruses

**DOI:** 10.1186/1751-0147-54-63

**Published:** 2012-11-06

**Authors:** Manabu Nemoto, Hiroshi Tsunemitsu, Harutaka Murase, Yasuo Nambo, Shinsuke Sato, Yasuhiro Orita, Hiroshi Imagawa, Hiroshi Bannai, Koji Tsujimura, Takashi Yamanaka, Tomio Matsumura, Takashi Kondo

**Affiliations:** 1 Epizootic Research Center, Equine Research Institute, Japan Racing Association, 1400-4 Shiba, Shimotsuke, Tochigi, 329-0412, Japan; 2 Research Team for Viral Diseases, National Institute of Animal Health, 3-1-5 Kannondai, Tsukuba, Ibaraki, 305-0856, Japan; 3 Hidaka Training and Research Center, Japan Racing Association, 535-13 Nishicha, Urakawa-cho, Urakawa-gun, Hokkaido, 057-0171, Japan; 4 Hidaka Agriculture Mutual Aid Association, 8-71 Hokusei-cho, Niikappu-cho, Niikappu-gun, Hokkaido, 059-2403, Japan

**Keywords:** Equine rotavirus, Vaccine, G3BP[12], G14P[12]

## Abstract

**Background:**

Both the G3P[12] and the G14P[12] type of equine group A rotavirus (RVA) have recently become predominant in many countries, including Japan. G3 types are classified further into G3A and G3B. The G3A viruses have been circulating in Europe, Australia, and Argentina, and the G3B viruses have been circulating in Japan. However, only an inactivated vaccine containing a single G3BP[12] strain is commercially available in Japan. To assess the efficacy of the current vaccine against recently circulating equine RVA strains, we examined antibody responses in pregnant mares to recent G3BP[12] and G14P[12] strains by virus neutralization test.

**Findings:**

After vaccination in five pregnant mares, the geometric mean serum titers of virus-neutralizing antibody to recent G3BP[12] strains increased 5.3- to 7.0-fold and were similar to that against homologous vaccine strain. Moreover, antibody titers to recent G14P[12] strains were also increased 3.0- to 3.5-fold.

**Conclusions:**

These results suggest that inoculation of mares with the current vaccine should provide foals with virus-neutralizing antibodies against not only the G3BP[12] but also the G14P[12] RVA strain via the colostrum.

## Findings

Group A rotavirus (RVA) is a non-enveloped virus belonging to the genus *Rotavirus* in the family Reoviridae. RVA has 11 double-stranded RNA genome segments [1]. Equine RVA infection is a major cause of diarrhea in foals up to 3 months old [2]. RVA has two outer capsid proteins, VP7 and VP4, which independently elicit the formation of neutralizing antibodies and induce protective immunity. These proteins are used to classify RVAs into G (for glycoprotein) and P (for protease-sensitive) types [1]. In addition, a whole-genome classification system based on nucleotide sequences has been proposed by using the following formula: Gx-P[x]-Ix-Rx-Cx-Mx-Ax-Nx-Tx-Ex-Hx [3].

Either the G3P[12] or the G14P[12] type of equine RVA, or mixed infections, have recently become predominant in many countries [4-8]. G3 types are categorized into two antigenic subtypes, G3A and G3B, on the basis of cross-neutralization assays and their different reactivity with a panel of monoclonal antibodies [9]. The G3A viruses have been circulating in Europe, Australia, and Argentina [5-7], and the G3B viruses have been circulating in Japan [10,11]. Recently, whole genome analysis has revealed that the genotype constellation is highly conserved among G3/G14 equine RVA strains in Argentina, Ireland, and South Africa (G3/G14-P[12]-I2/I6-R2-C2-M3-A10-N2-T3-E2/E12-H7) [12].

A few inactivated vaccines have been developed for the prevention of diarrhea induced by equine RVA. These vaccines are administered intramuscularly to pregnant mares, and their newborn foals obtain passive immunity via the colostrum. RVA/Horse-tc/GBR/H-2/1976/G3AP[12] (H-2) has been used as a vaccine strain in the USA [13], the United Kingdom, and Ireland [5], and three RVA strains (H-2, RVA/Simian-tc/ZAF/SA11/1958/G3P[2], and RVA/Cow-tc/USA/NCDV-Lincoln/1967/G6P[1]) have been used for vaccination in Argentina [14]. Vaccines containing the H-2 strain increase antibody titers to a homologous strain [13] and tend to reduce rotavirus diarrhea in the field [13,14]. In Japan, only G3BP[12] viruses circulated until the early 1990s [15]. In light of this epidemic situation, an inactivated vaccine containing the strain RVA/Horse-tc/JPN/HO-5/1982/G3BP[12] (HO-5) was developed for the prevention of diarrhea induced by equine RVA in Japan [16,17]. The Japanese vaccine has been commercially available since 2001 (Nisseiken Co., Ltd., Tokyo, Japan). Experimental infection with a homologous virus has shown that this vaccine effectively reduces clinical signs [17].

Although G3BP[12] and G14P[12] viruses have become predominant in Japan, the effectiveness of the existing vaccine against these recently circulating viruses is unclear. In this study, we used virus neutralization testing to examine the antibody responses of vaccinated pregnant mares against recently circulating equine RVA strains in Japan to evaluate the efficacy of the current vaccine.

In 2010, fecal samples were obtained from four diarrhea-affected foals in the Hidaka district of Hokkaido, Japan. All of the foals had been reared on different farms. Virus isolation was performed by using MA-104 [18] and Caco-2 [19] cells, as described previously, with a slight modification. The G type of isolated viruses was determined by semi-nested RT-PCR specific for G3 or G14 types [11], and the P type was determined by RT-LAMP specific for the P[12] type [20]. The RT-PCR products of isolated viruses were amplified by using the primers Beg9/End9 [21] and Con3/Con2 [22] and sequenced commercially at the Dragon Genomics Center (Takara Bio Inc, Mie, Japan). Phylogenetic analysis was conducted with MEGA software Version 4.0 [23]. Phylogenetic trees, based on the VP7 and VP4 gene sequences, were constructed by using the neighbor-joining method. Statistical analyses of the trees were performed by employing the bootstrap test (1000 replicates) for multiple alignments.

Four equine RVA strains were isolated from the fecal samples of foals affected with diarrhea. Two G3P[12] strains isolated in MA-104 cells were designated as strains RVA/Horse-tc/JPN/No.1/2010/G3BP[12] (No. 1/2010) and RVA/Horse-tc/JPN/No. 13/2010/G3BP[12] (No. 13/2010), and two G14P[12] strains isolated in Caco-2 cells were designated as strains RVA/Horse-tc/JPN/No. 24/2010/G14P[12] (No. 24/2010) and RVA/Horse-tc/JPN/No. 50/2010/G14P[12] (No. 50/2010). The accession numbers registered in GenBank were as follows: partial sequences of the VP7 gene, No. 1/2010 (AB703265), No. 13/2010 (AB703266), No. 24/2010 (AB703267), and No. 50/2010 (AB703268); and partial sequences of the VP4 gene, No. 1/2010 (AB703269), No. 13/2010 (AB703270), No. 24/2010 (AB703271), and No. 50/2010 (AB703272).

**Figure 1:**
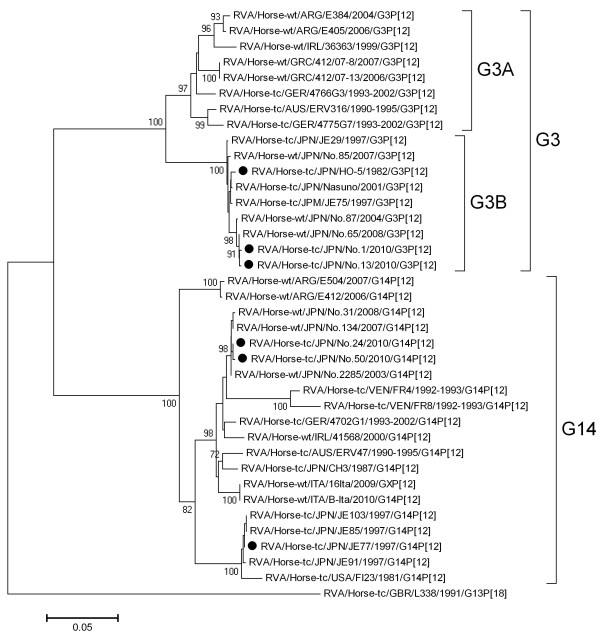
**Phylogenetic analysis of the VP7 gene of RVA strains G3BP[12] and G14P[12].** Rotavirus strains were denominated as reported previously [24]. The nucleotide sequences (nucleotides 104 to 906) of the VP7 gene were used to conduct a phylogenetic analysis. Black dots indicate the VP7 genes of equine RVA strains used in the virus-neutralizing test. Genome positions correspond to that of RVA/Horse-tc/JPN/HO-5/1982/G3P[12] (GenBank accession number AB046464). RVA/Horse-wt/GBR/L338/1991/G13P[18] is included as an outgroup. Percentage bootstrap support is indicated by the value at each node; values of <70 have been omitted.

In the phylogenetic tree of VP7 (Figure 1), strains No. 1/2010 and No. 13/2010 were clustered into the G3B type, together with the homologous strain HO-5. The nucleotide sequence (nucleotides 104 to 906) identity among these strains was 98.9% to 99.8%. Strains No. 24/2010 and No. 50/2010 were clustered into the G14 type known to have circulated from 2003 to 2008 and were classified differently from the JE77-like G14 strains circulating in the late 1990s. Because the JE77-like G14 strains are no longer epidemic, they may have disappeared and been replaced by these newer G14-type strains. The level of nucleotide sequence identity between the JE77 strain and No. 24/2010 was 93.4%, and that between JE77 and No. 50/2010 was 94.0%.

**Figure 2:**
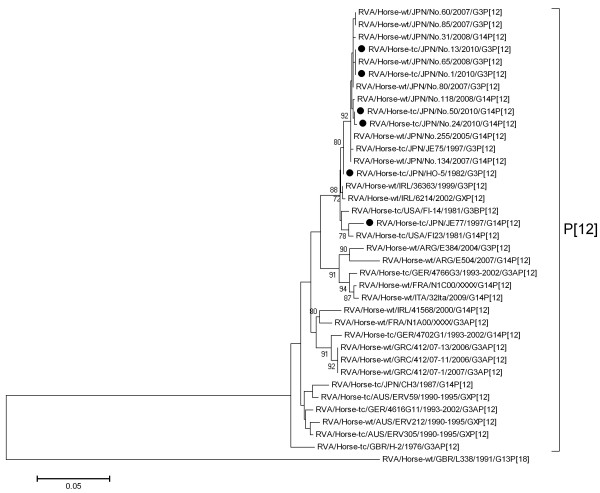
**Phylogenetic analysis of the VP4 gene of RVA strains G3BP[12] and G14P[12].** The nucleotide sequences (nucleotides 114 to 755) of the VP4 gene were used to conduct a phylogenetic analysis. Black dots indicate the VP4 genes of the equine RVA strains used in the virus-neutralizing test. Genome positions correspond to that of RVA/Horse-tc/JPN/HO-5/1982/G3P[12] (Genbank accession number AB046471). RVA/Horse-wt/GBR/L338/1991/G13P[18] is included as an outgroup. Percentage bootstrap support is indicated by the value at each node; values of <70 have been omitted.

In the phylogenetic tree of VP4, all four strains belonged to the P[12] type and were located in the same cluster as the HO-5 strain and the equine RVA strains circulating from 2003 to 2008, irrespective of their G types (Figure 2). These results are in accord with the data that we have investigated using fecal samples collected from 2003 to 2008 [10].

In 2010 to 2011, five pregnant mares (4 to 11 years old, average age 8.2 years) that had no history of vaccination against equine RVA and had been reared at the Hidaka Training Farm of the Japan Racing Association, in the Hidaka district of Hokkaido, were twice inoculated with 2 ml of inactivated equine RVA vaccine (Nisseiken Co., Ltd., Tokyo, Japan.) at an interval of 4 to 8 weeks. These mares delivered within 3 to 6 weeks after the second vaccination. Paired sera were collected from the mares 0 to 7 days before the first vaccination and at parturition.

Virus neutralization tests were performed by using the fluorescent focus neutralization test and MA-104 cells, as described previously [25]. Virus-neutralizing antibody titers were expressed as the reciprocal of the highest serum dilution that resulted in an 80% or greater reduction in fluorescent foci. RVA strains used as reference viruses were HO-5 and RVA/Horse-tc/JPN/JE77/1997/G14P[12] (JE77).

The virus-neutralizing antibody titers of the horse sera are shown in Table 1. After vaccination, the geometric mean titers against the homologous strain HO-5 (G3BP[12]) and against No. 1/2010 (G3BP[12]), No. 13/2010 (G3BP[12]), JE77 (G14P[12]), No. 24/2010 (G14P[12]), and No. 50/2010 (G14P[12]) were 735, 970, 970, 320, 243, and 243, respectively. The immune sera neutralized No. 1/2010 and No. 13/2010 at titers similar to those that neutralized the homologous strain. Because the VP7 and VP4 genes are highly conserved among HO-5, No. 1/2010, and No. 13/2010 (Figure 1), we expected that the antigenicity of these strains was conserved. In contrast, the geometric mean antibody titers to the G14P[12] viruses were lower than that to the homologous virus, although they were 3.0- to 3.5-fold increased after vaccination. Browning *et al.* have reported that mares immunized with a single-type (G6) inactivated bovine rotavirus vaccine develop antibodies in the serum and milk not only to the G6 type but also to the G2 and the G3 type [26]. Such a heterotypic response has been also reported in adult cows [27,28]. Our present result indicates that the G3BP[12] vaccine would also induce virus-neutralizing antibody against heterologous G14P[12] viruses. In addition to the immunogenicity of the G3BP[12] vaccine, investigations into that of the G3AP[12] vaccine is needed to understand vaccine efficacy and future vaccine design.

**Table 1:** Virus-neutralizing titers in mares before and after immunization with the inactivated vaccine

**Virus**^a^	**G and P type**	**Titer pre-vaccination**						**Titer post-vaccination**					
		**Horse no.**						**Horse no.**					
		**1**	**2**	**3**	**4**	**5**	**GMT**^b^	**1**	**2**	**3**	**4**	**5**	**GMT**
HO-5	G3BP[12]	160	160	40	80	160	106	1280	640	640	640	640	735
No. 1/2010	G3BP[12]	160	320	160	40	160	139	2560	1280	1280	320	640	970
No. 13/2010	G3BP[12]	320	320	160	80	160	184	2560	1280	1280	320	640	970
JE77	G14P[12]	320	80	80	40	80	92	1280	160	640	160	160	320
No. 24/2010	G14P[12]	160	80	80	40	80	80	640	160	640	80	160	243
No. 50/2010	G14P[12]	160	80	80	40	80	80	640	320	320	80	160	243

^a^ See text for abbreviations.

^b^ Geometric mean titer.

Unfortunately, we could not obtain colostrum from the vaccinated mares or sera from their foals. However, because antibody titers against equine RVA in the sera of pregnant mares are closely related to those of their colostrum and their foals’ sera [17], it should be possible to deduce the immune status of the foals from the serum antibody titers of their mares. Therefore, this study shows that the current vaccine is likely to provide foals with antibodies against recent G3BP[12] RVA strains at the same level as those against the vaccine strain. Of note, in the late 1990s it was reported that the current vaccine reduced the duration and clinical signs of diarrhea caused by G14 viruses [16]. Mares immunized with the current vaccine neutralized strains No. 24/2010 and No. 50/2010 at titers similar to those at which they neutralized strain JE77 isolated in the late 1990s (Table 1). These results suggest that inoculation with the current vaccine also appears to provide foals with virus-neutralizing antibodies against recent G14P[12] RVA strains via the colostrum. We used only a small number of samples in this study, and the results need to be confirmed with a large number of samples. Virus challenge studies in foals might be needed to clarify the protective efficacy of the vaccine against recent G14P[12] strains.

## Competing interests

The authors declare that they have no competing interests.

## Authors’ contributions

MN outlined the design of the study, performed the experiments, and drafted the manuscript. HT, HI, TY, TM, and TK participated in the design of the study and interpretation of the data and helped to draft the manuscript. HM, YN, SS, and YO carried out the clinical observations of horses and the sample collections. HB and KT performed several experiments. All authors read and approved the final manuscript.

## References

[B1] Estes MK Kapikian AZ Knipe DM, Howley PM Rotaviruses Fields Virology 2007 5 Philadelphia: Lippincott Williams & Wilkins 1917 1974

[B2] Imagawa H Sekiguchi K Anzai T Fukunaga Y Kanemaru T Ohishi H Higuchi T Kamada M Epidemiology of equine rotavirus infection among foals in the breeding region J Vet Med Sci 1991 53 1079 1080 10.1292/jvms.53.1079 1790219

[B3] Matthijnssens J Ciarlet M Heiman E Arijs I Delbeke T McDonald SM Palombo EA Iturriza-Gomara M Maes P Patton JT Full genome-based classification of rotaviruses reveals a common origin between human Wa-Like and porcine rotavirus strains and human DS-1-like and bovine rotavirus strains J Virol 2008 82 3204 3219 10.1128/JVI.02257-07 18216098 PMC2268446

[B4] Elschner M Schrader C Hotzel H Prudlo J Sachse K Eichhorn W Herbst W Otto P Isolation and molecular characterisation of equine rotaviruses from Germany Vet Microbiol 2005 105 123 129 10.1016/j.vetmic.2004.10.010 15627523

[B5] Collins PJ Cullinane A Martella V O'Shea H Molecular characterization of equine rotavirus in Ireland J Clin Microbiol 2008 46 3346 3354 10.1128/JCM.00995-08 18716232 PMC2566120

[B6] Ntafis V Fragkiadaki E Xylouri E Omirou A Lavazza A Martella V Rotavirus-associated diarrhoea in foals in Greece Vet Microbiol 2010 144 461 465 10.1016/j.vetmic.2010.01.020 20197218

[B7] Garaicoechea L Mino S Ciarlet M Fernandez F Barrandeguy M Parreno V Molecular characterization of equine rotaviruses circulating in Argentinean foals during a 17-year surveillance period (1992–2008) Vet Microbiol 2011 148 150 160 10.1016/j.vetmic.2010.08.032 20943330

[B8] Monini M Biasin A Valentini S Cattoli G Ruggeri FM Recurrent rotavirus diarrhoea outbreaks in a stud farm, in Italy Vet Microbiol 2011 149 248 253 10.1016/j.vetmic.2010.11.007 21129862

[B9] Browning GF Chalmers RM Fitzgerald TA Snodgrass DR Evidence for two serotype G3 subtypes among equine rotaviruses J Clin Microbiol 1992 30 485 491 1371520 10.1128/jcm.30.2.485-491.1992 PMC265082

[B10] Nemoto M Tsunemitsu H Imagawa H Hata H Higuchi T Sato S Orita Y Sugita S Bannai H Tsujimura K Molecular characterization and analysis of equine rotavirus circulating in Japan from 2003 to 2008 Vet Microbiol 2011 152 67 73 10.1016/j.vetmic.2011.04.016 21565456

[B11] Tsunemitsu H Imagawa H Togo M Shouji T Kawashima K Horino R Imai K Nishimori T Takagi M Higuchi T Predominance of G3B and G14 equine group A rotaviruses of a single VP4 serotype in Japan Arch Virol 2001 146 1949 1962 10.1007/s007050170044 11722016 PMC7087255

[B12] Matthijnssens J Mino S Papp H Potgieter C Novo L Heylen E Zeller M Garaicoechea L Badaracco A Lengyel G Complete molecular genome analyses of equine rotavirus A strains from different continents reveal several novel genotypes and a largely conserved genotype constellation J Gen Virol 2012 93 866 875 10.1099/vir.0.039255-0 22190012

[B13] Powell DG Dwyer RM Traub-Dargatz JL Fulker RH Whalen JW Jr Srinivasappa J Acree WM Chu HJ Field study of the safety, immunogenicity, and efficacy of an inactivated equine rotavirus vaccine J Am Vet Med Assoc 1997 211 193 198 9227750

[B14] Barrandeguy M Parreno V Lagos Marmol M Pont Lezica F Rivas C Valle C Fernandez F Prevention of rotavirus diarrhoea in foals by parenteral vaccination of the mares: field trial Dev Biol Stand 1998 92 253 257 9580371

[B15] Imagawa H Tanaka T Sekiguchi K Fukunaga Y Anzai T Minamoto N Kamada M Electropherotypes, serotypes, and subgroups of equine rotaviruses isolated in Japan Arch Virol 1993 131 169 176 10.1007/BF01379088 8392320

[B16] Imagawa H Kato T Tsunemitsu H Tanaka H Sato S Higuchi T Field study of inactivated equine rotavirus vaccine J Equine Sci 2005 16 35 44 10.1294/jes.16.35

[B17] Imagawa H Wada R Sugita S Fukunaga Y Wernery U, Wade JF, Mumford JA, Kaaden O-R Passive immunity in foals of mares immunized with inactivated equine rotavirus vaccine Equine Infectious Disease VIII 1999 Newmarket: R & W Publications 201 205

[B18] Imagawa H Wada R Hirasawa K Akiyama Y Oda T Isolation of equine rotavirus in cell cultures from foals with diarrhea Jpn J Vet Sci 1984 46 1 9 10.1292/jvms1939.46.1 6330417

[B19] Shinozaki K Yamanaka T Tokieda M Shirasawa H Simizu B Isolation and serial propagation of human group C rotaviruses in a cell line (CaCo-2) J Med Virol 1996 48 48 52 10.1002/(SICI)1096-9071(199601)48:1<48::AID-JMV8>3.0.CO;2-L 8825710

[B20] Nemoto M Imagawa H Tsujimura K Yamanaka T Kondo T Matsumura T Detection of equine rotavirus by reverse transcription loop-mediated isothermal amplification (RT-LAMP) J Vet Med Sci 2010 72 823 826 10.1292/jvms.09-0446 20160420

[B21] Gouvea V Glass RI Woods P Taniguchi K Clark HF Forrester B Fang ZY Polymerase chain reaction amplification and typing of rotavirus nucleic acid from stool specimens J Clin Microbiol 1990 28 276 282 2155916 10.1128/jcm.28.2.276-282.1990 PMC269590

[B22] Gentsch JR Glass RI Woods P Gouvea V Gorziglia M Flores J Das BK Bhan MK Identification of group A rotavirus gene 4 types by polymerase chain reaction J Clin Microbiol 1992 30 1365 1373 1320625 10.1128/jcm.30.6.1365-1373.1992 PMC265294

[B23] Tamura K Dudley J Nei M Kumar S MEGA4: Molecular Evolutionary Genetics Analysis (MEGA) software version 4.0 Mol Biol Evol 2007 24 1596 1599 10.1093/molbev/msm092 17488738

[B24] Matthijnssens J Ciarlet M McDonald SM Attoui H Banyai K Brister JR Buesa J Esona MD Estes MK Gentsch JR Uniformity of rotavirus strain nomenclature proposed by the Rotavirus Classification Working Group (RCWG) Arch Virol 2011 156 1397 1413 10.1007/s00705-011-1006-z 21597953 PMC3398998

[B25] Ojeh CK Jiang BM Tsunemitsu H Kang SY Weilnau PA Saif LJ Reactivity of monoclonal antibodies to the 41-kilodalton protein of porcine group C rotavirus with homologous and heterologous rotavirus serogroups in immunofluorescence tests J Clin Microbiol 1991 29 2051 2055 1663522 10.1128/jcm.29.9.2051-2055.1991 PMC270259

[B26] Browning GF Chalmers RM Sale CS Fitzgerald TA Snodgrass DR Homotypic and heterotypic serum and milk antibody to rotavirus in normal, infected and vaccinated horses Vet Microbiol 1991 27 231 244 10.1016/0378-1135(91)90150-E 1715620 PMC7117508

[B27] Snodgrass DR Fitzgerald TA Campbell I Browning GF Scott FM Hoshino Y Davies RC Homotypic and heterotypic serological responses to rotavirus neutralization epitopes in immunologically naive and experienced animals J Clin Microbiol 1991 29 2668 2672 1723075 10.1128/jcm.29.11.2668-2672.1991 PMC270403

[B28] Brussow H Walther I Fryder V Sidoti J Bruttin A Cross-neutralizing antibodies induced by single serotype vaccination of cows with rotavirus J Gen Virol 1988 69 1647 1658 10.1099/0022-1317-69-7-1647 2839600

